# Identification and Expression Analysis of *UPS* Gene Family in Potato

**DOI:** 10.3390/genes15070870

**Published:** 2024-07-02

**Authors:** Wenyue Huang, Yifei Lu, Bi Ren, Fuchun Zeng, Yongjian Liu, Liming Lu, Liqin Li

**Affiliations:** College of Agronomy, Sichuan Agricultural University, Chengdu 611130, China; huangwenyue1106x@163.com (W.H.); sarklu@126.com (Y.L.); renbi1232021@163.com (B.R.); zengfuchun78@163.com (F.Z.); yjliu2010@sohu.com (Y.L.); luliming@sicau.edu.cn (L.L.)

**Keywords:** ureide permease, bioinformatics analysis, potato, gene expression, abiotic stress

## Abstract

Ureide permeases (UPSs) mediate the transport of ureides, including allantoin and allantoate, which act as nitrogen-transporting compounds in plants and have recently been found to play a role in cellular signaling. To date, UPSs have not been reported in potato, and their identification is important for further function studies and for understanding molecular mechanisms of plant adverse responses. Based on potato genomic data, we identified 10 *StUPS* genes in potato (*Solanum tuberosum* L.). Then, we conducted a comprehensive study of the identified *StUPS* genes using bioinformatics methods. Genome phylogenetic and genomic localization analyses revealed that *StUPSs* can be classified into four categories, are highly homologous to *Arabidopsis thaliana* UPS members, and are distributed on three chromosomes. The six *StUPS* genes were investigated by RT–qPCR, and the findings indicated that all of these genes are involved in the response to several stresses, including low nitrogen, cold, ABA, salt, H_2_O_2,_ and drought. This study establishes a strong theoretical framework for investigating the function of potato *UPS* genes, as well as the molecular mechanisms underlying the responses of these genes to various environmental stresses.

## 1. Introduction

Transporter proteins play an important role in cellular material transport, enabling various molecules to move across biological membranes, and play a crucial role in nutrient uptake, transportation, and distribution [1,2]. Ureide permeases (UPSs) are a class of transporter proteins that mediate the transport of ureides in plants. Ureides are derived from the catabolic pathway of purines, including allantoin and allantoate, which are nitrogen-rich organic compounds [3,4,5,6,7]. In animals, uric acid is the end product of purine metabolism. In plants, uric acid can be further broken down into allantoin (peroxisomes). In the endoplasmic reticulum, allantoin can be further decomposed into allantoate, which is further broken down to form carbon dioxide, NH_4_^+^, and eventually glyoxylate [8]. Nitrogen (N) is an indispensable major element for plant growth, and plays an essential role in the formation of potato organs and material metabolism. N also determines the yield of potato tubers. The efficient use of N can significantly increase dry matter accumulation and the economic benefits of potato. Potato is one of the world’s four major food crops; however, N use efficiency tends to be low in potato. N utilization in plants includes the processes of N uptake, transportation, and reutilization [1]. Ureides are generally synthesized in roots, and UPS is involved in the transport of ureides from roots to aboveground parts via the xylem and from source leaves to reservoir organs such as seeds via the phloem [2,9,10], thus affecting the accumulation and distribution of ureides in various organs and participating in N metabolism in plants [7,9,10,11]. In mature roots, several UPS family members, including PvUPS1 from French beans and GmUPS1-1 and GmUPS1-2 from soybeans, mediate allantoin symplasmic transfer and xylem loading [6,7,12]. PvUPS1 may also play a role in allantoin phloem loading [10].

Ureides were initially widely studied in legumes as a product of rhizomatous N fixation, and the primary form of N is transported over long distances in legumes [13]. In non-leguminous plants, amino acids are usually considered the primary form of N for long-distance transport, but recently, the importance of ureides in N metabolism has been recognized [14,15,16]. The ureide content is affected by a variety of factors in plants, including environmental factors, species, growth, and development [17]. In *Arabidopsis thaliana*, *AtUPS5L* expression is specifically up-regulated under salt stress, suggesting that AtUPS5L plays a dominant role in mediating allantoin transport [18]. When rice is under high-N conditions, OsUPS1 is activated, thereby mediating allantoin transport to its target site. In contrast, transport is inhibited under low-nitrogen (LN) conditions [15]. A relatively high allantoin content in leaves and grains is positively correlated with increased N utilization efficiency in wheat varieties [14]. However, recent studies have also demonstrated that allantoin accumulation increases plant tolerance to different abiotic stresses [19,20,21].

Currently, the functions of UPS family members in potato have not been fully characterized. Considering the importance of the UPS family and the financial advantages of potatoes, the identification and analysis of the UPS family are worthwhile. In this study, ten UPS members were identified in potato via bioinformatics methods. Our study revealed that the *UPS* genes in potato and *Arabidopsis* are highly homologous, indicating that they may serve similar functions under different stresses. Furthermore, to determine whether *StUPS* genes play a role in how plants respond to abiotic stresses (salt, drought, ABA, H_2_O_2_, cold, and low nitrogen), we selected six genes (*StUPS3*, *StUPS4*, *StUPS6*, *StUPS7*, *StUPS8*, and *StUPS9*) and examined their expression using RT–qPCR. These findings suggest that *StUPS* genes play a crucial role in plant response to abiotic stresses. This study not only investigated the function of *StUPS* genes, but also created the groundwork for selecting and breeding highly resistant potato cultivars.

## 2. Materials and Methods

### 2.1. Plant Materials and Growing Conditions

In this study, Chuanyu No.10 potato histocultured seedlings provided by the Potato Research and Development Center, College of Agriculture, Sichuan Agricultural University, were used as experimental materials. Histocultured seedlings grown on an MS medium for 20 days were removed and selected for uniformity and good growth, after which the roots were washed with water and then exposed to stress treatment (Table S1). Samples were taken at 0 h and 6 h of treatments, and then stored at −80 °C.

### 2.2. Identification of Potato UPS Family Members

To identify all members of the potato *UPS* gene family, we obtained the HMM file (PF07168) from the Pfam database (http://pfam-legacy.xfam.org/, accessed on 9 January 2024) by searching for keywords (ureide permease) [22]. Then, the file was submitted to the HMM database (https://www.ebi.ac.uk/Tools/hmmer/search/hmmsearch, accessed on 9 January 2024) to retrieve sequences containing UPS structural domains [23]. Finally, incomplete and erroneous sequences were deleted through the Conserved Structural Domain Database (CDD) on the National Center for Biotechnology Information (NCBI) online website.

### 2.3. Physicochemical Properties, Transmembrane Structure, and Subcellular Localization of Potato UPS Proteins

The physicochemical features of potato UPS proteins were evaluated utilizing the internet website Expasy-ProtParam (https://web.expasy.org/protparam/, accessed on 9 January 2024) [24]. The transmembrane structure of the UPS protein was analyzed using the online site TMHMM 2.0 (https://services.healthtech.dtu.dk/services/TMHMM-2.0/, accessed on 21 June 2024) [25]. Subcellular localization of potato UPS proteins was predicted using the online website WoLF PSORT (https://wolfpsort.hgc.jp/, accessed on 9 January 2024) [26].

### 2.4. Phylogenetic Analysis of UPS Proteins

The UPS phylogenetic analysis included 60 UPS amino acid sequences from *Arabidopsis* and potato. MEGA 7.0 software was implemented for phylogenetic analysis [27], and an evolutionary tree was constructed with the neighbor-joining method to infer the evolutionary history. Multiple amino acid sequences of UPS proteins from *Arabidopsis* and potato were aligned using ClustalW included in MEGA 7.0 software. Parameters were set using deletion data, P-distance modeling, and Bootstrap tests with 1000 paired deletion instances. Default values were used for other parameter settings. Then, the evolutionary tree was landscaped using the internet site Interactive Tree of Life (ITOL, https://itol.embl.de/, accessed on 9 January 2024) [28].

### 2.5. Analysis of UPS Motifs and Gene Structures in Potato

We obtained the potato genome annotation file (gtf) from the Ensembl Plants database (https://plants.ensembl.org/index.html, accessed on 10 January 2024) [29]. Then, the gene structure information of *StUPSs* was extracted from the gtf file by using TBtools (v2.042), and the gene structures were visualized in TBtools [30]. The motifs of the candidate genes were predicted using TBtools (v2.042), with the base sequence length ranging from 6 to 50 and the number of base sequences set to 10.

### 2.6. Analysis of Chromosomal Location

Based on the potato genome annotation file, information on the start and end positions of the *StUPS* genes on the corresponding chromosomes was extracted, and then, the positional distribution of the *StUPS* gene family on the chromosomes was visualized utilizing TBtools (v2.042).

### 2.7. Collinearity of UPS Family in Potato

We obtained the potato genome file from the Ensembl Plants database (https://plants.ensembl.org/index.html, accessed on 10 January 2024) [29]. Based on the downloaded potato genome files and genome annotation files, we performed collinearity analysis of the *StUPS* gene family with TBtools. Furthermore, to investigate the evolutionary relationships of *UPS* in various species, we visualized the covariance of UPS families in *Arabidopsis*, potato, and tomato using MCS canX (TBtools v2.042).

### 2.8. Expression Pattern Analysis of the UPS Gene Family

From the Spud DB database (http://spuddb.uga.edu/index.shtml, accessed on 11 January 2024), we were able to obtain transcriptome data for the potato [31]. The tissue expression patterns of potato *UPS* family members and their functions under stress conditions were analyzed by using TBtools software (v2.042) with cluster analysis and evolutionary relationships.

### 2.9. Expression Verification of Screened UPS Genes

Based on the clustered heatmap of *UPS* genes under various abiotic stresses, we screened six genes highly responsive to abiotic stresses and designed their primers (Table S2). A control group and six stress treatment groups were established. The treatment groups included low nitrogen (3.75 mmol/L), cold (4 °C), ABA (1 μmol/L), drought (5% PEG-6000), salt (200 mmol/L), and H_2_O_2_ (10 mmol/L). The control group received no stress treatment. The TRIzol technique was utilized to extract total RNA, and the Servicebio Reverse Transcription Kit was employed for synthesizing cDNA. Hieff^®^ qPCR SYBR^®^ Green Master Mix (no. 11201ES03) was applied for real-time fluorescence quantitative PCR reaction. We used elongation factor 1-α (ef1α) as a reference gene for RT-qPCR [32]. The reaction system was 10.0 μL: 5.0 μL SYBR^®^ Green Master Mix, 1 μL cDNA, 0.2 μL each of forward and reverse primers, and 3.6 μL ddH_2_O. The reaction conditions were as follows: pre−denaturation at 95 °C for 5 min, denaturation at 95 °C for 10 s, and annealing at 60 °C for 30 s for 40 cycles. Next, we made use of the 2^−∆∆CT^ method to calculate the relative expression of the genes [33].

## 3. Results

### 3.1. Identification of the Potato UPS Family Members

We utilized the HMM database to search for sequences containing UPS structural domains in the potato whole-protein database with HMM files as the search criteria. Finally, we identified 10 potato *UPS* genes and renamed them *StUPS1* to *StUPS10*.

### 3.2. Physicochemical Properties, Transmembrane Structure, and Subcellular Localization of UPS Proteins

**Figure 1:**
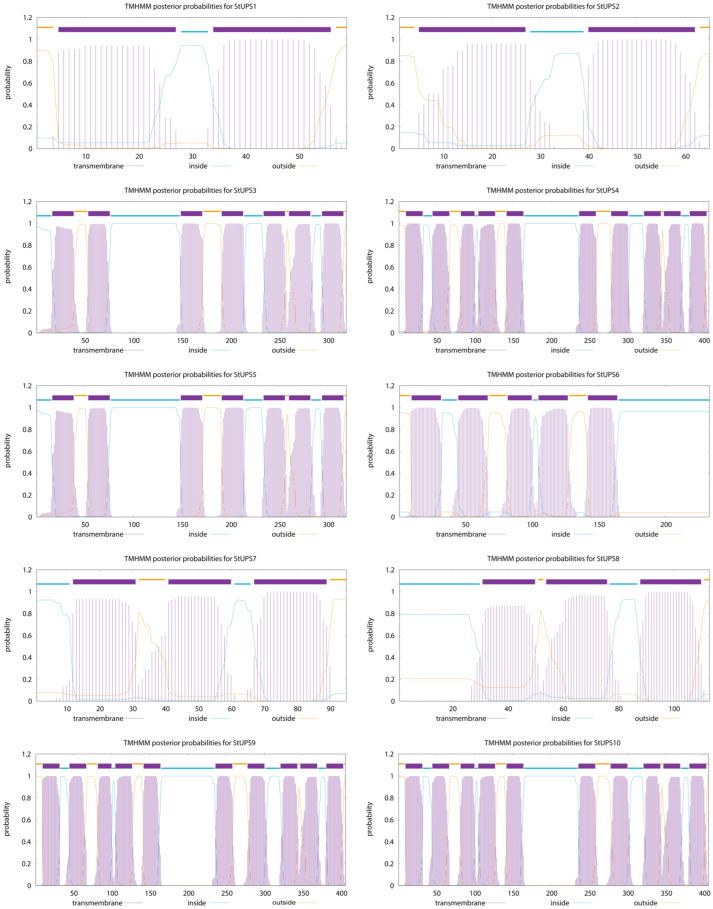
Analysis of the transmembrane structure of the StUPS proteins.

Physicochemical property and subcellular localization analyses of the identified potato UPS family members yielded the following results. In summary, the StUPS protein sequence lengths ranged from 59 to 407 amino acids (aa), the molecular weights ranged from 6.7 (StUPS1) to 44.2 (StUPS9) kDa, and the predicted isoelectric points ranged from 6.13 (StUPS6) to 10.55 (StUPS1). Instability indices greater than 40 are defined as unstable proteins and less than 40 as stable proteins; the grand average of hydrophilicity (GRAVY) positive values represents hydrophobicity, while negative values represent hydrophilicity. In addition, according to the predicted results, all proteins were defined as hydrophobic and stabilizing proteins except for StUPS1, which was an unstable protein. By analyzing the transmembrane structure of UPS proteins (Figure 1), the results show that all UPS proteins are transmembrane proteins. Subcellular localization revealed that three StUPS proteins were distributed in each of the vacuole and the plasma membrane, two were located extracellularly, and one was in each of the endoplasmic reticulum and chloroplasts (Table 1).

### 3.3. Phylogenetic Analysis of UPS Proteins

**Figure 2:**
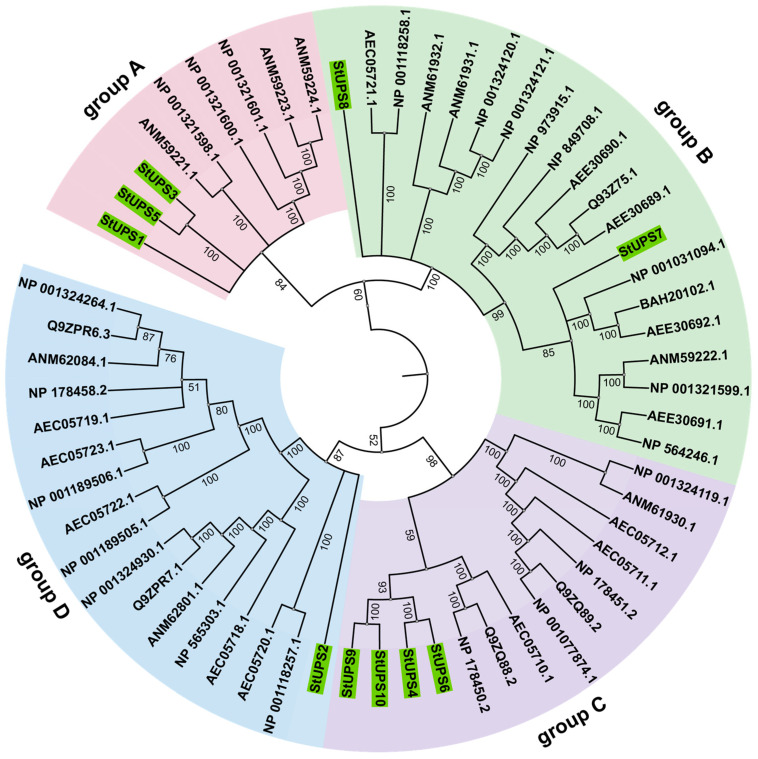
Phylogenetic tree of UPS proteins in potato and *Arabidopisis thaliana.* Groups A–D are distinguished by different color markings. UPS in potato highlighted with green markers.

We constructed a phylogenetic tree with the sequences of the UPS proteins from *Arabidopsis* and potato to evaluate the classification and evolutionary features of UPS proteins (Figure 2). A total of 60 sequences, including 50 AtUPSs and 10 StUPSs, were classified into four main groups based on homology. Group A contains three StUPS members (StUPS1, StUPS3, and StUPS5). Group B consists of two StUPS members, StUPS7/8. Group C includes four StUPS members, StUPS4, StUPS6, StUPS9, and StUPS10. Group D only contains StUPS2. Based on the above results, UPS members of the same subfamily may have similar biological functions.

### 3.4. Analysis of UPS Motifs and Gene Structures in Potato

**Figure 3:**
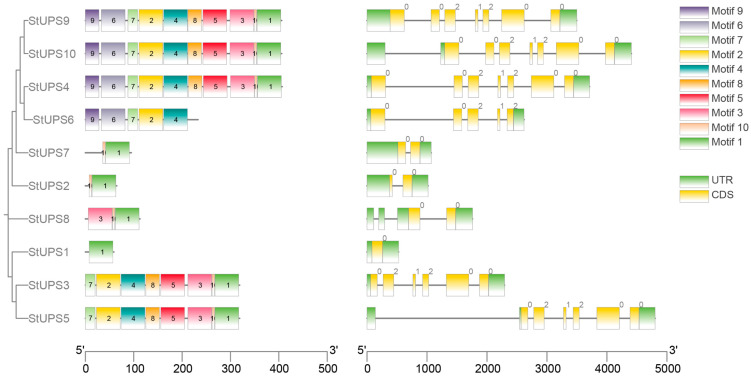
Motifs and gene structures of potato UPS family members. Left: motifs 1–10 are represented by different colored boxes. Right: black lines indicate introns; yellow boxes indicate CDS regions; green boxes indicate UTR regions.

TBtools visualized the distributions of StUPS protein motifs with the MEME file (Figure 3). The results showed that the number of motifs contained in each protein ranged from 1 to 10, and the motif composition was not the same among the members, but the motif composition, position, and number among the subfamilies showed covariance. Figure 3 demonstrates that, except for StUPS6, all UPS proteins have motif 1, and the majority of StUPS proteins contain motif 10.

During the evolution of a gene family, introns and exons play different roles in gene expression and regulation. Analysis of the number and distribution characteristics of introns and exons of the UPS family genes in potato (Figure 3) showed that there was a strong correlation between the structures of the gene family members during evolution, and that the members clustered together had similar structures. As illustrated in Figure 3, all *StUPSs* have exons and untranslated regions.

### 3.5. The Chromosomal Distribution of StUPS Genes

**Figure 4:**
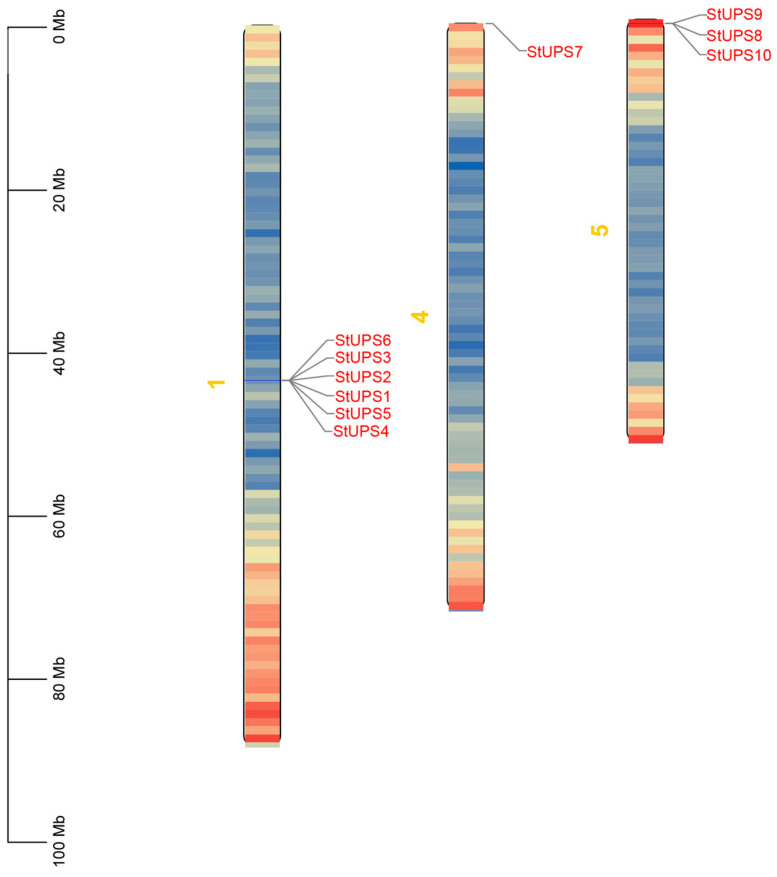
Chromosome location of *UPS* gene family in potato.

As displayed in Figure 4, the *StUPS* members were spread across three chromosomes: chr1, chr4, and chr5. Among them, six *StUPS* genes, *StUPS1*, *StUPS2*, *StUPS3*, *StUPS4*, *StUPS5*, and *StUPS6*, were located on chr1. Three *StUPS* genes were distributed on chr5 (*StUPS8*, *StUPS9*, and *StUPS10*), whereas only one *StUPS* gene (*StUPS7*) was found on chr4.

### 3.6. Collinearity Analysis of UPS Gene Family

**Figure 5:**
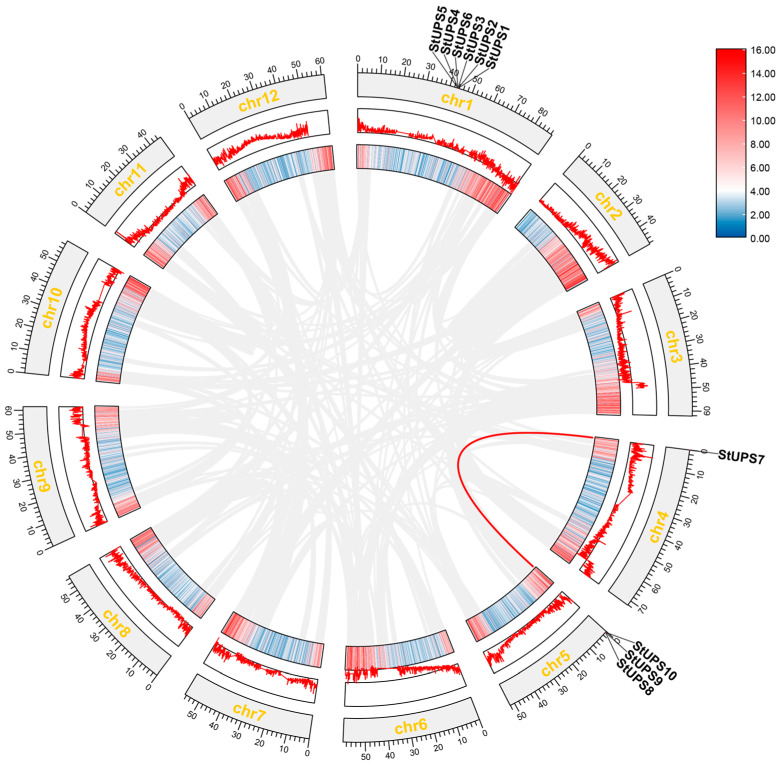
Collinearity analysis of the potato UPS family.

Plant evolution relies heavily on gene duplication events. We first performed collinearity analysis of *UPS* genes within potato species and analyzed all *StUPS* gene duplication events by using TBtools software to elucidate the duplication mechanism involved. One pair of *StUPS* gene duplication events, *StUPS7*/*StUPS9*, was detected; however, no tandem duplications were detected (Figure 5). Tandem duplication events are chromosomal regions within 200 kb that contain two or more family genes [34].

**Figure 6:**
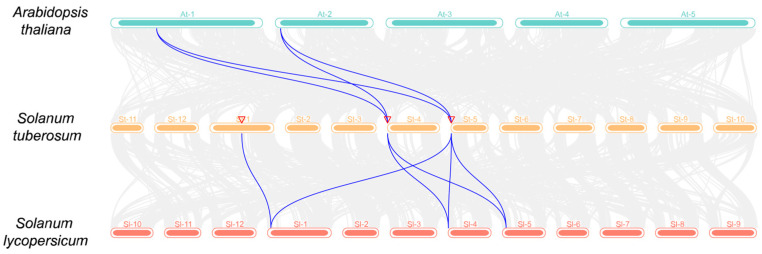
Collinearity analysis of the *UPS* gene families in *Arabidopsis*, potato, and tomato.

In addition, to explore the evolutionary relationships of the *UPS* gene family in different species, we analyzed the covariance among potato, *Arabidopsis*, and tomato. Four pairs of covariance between potato and *Arabidopsis* were found, as shown in Figure 6, whereas there were six pairs of covariance between potato and tomato, which is consistent with the species evolutionary relationships.

### 3.7. Expression Pattern Analysis of UPS Genes in Potato

**Figure 7:**
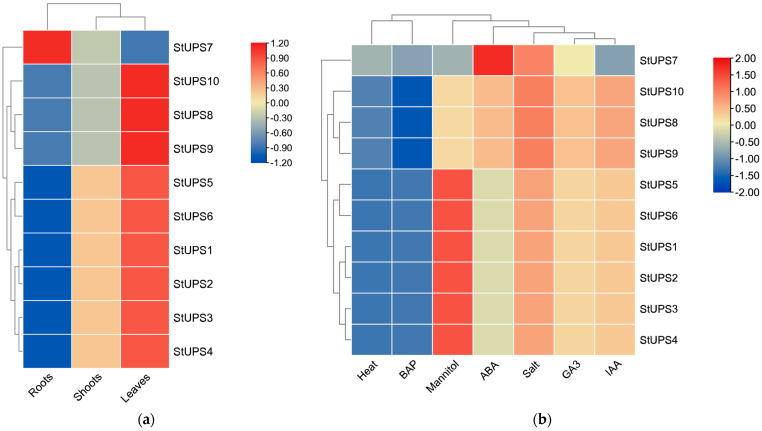
Expression profile of *UPS* genes in potato. (**a**) Expression profile in various tissues; (**b**) expression profile under various abiotic stresses.

To further investigate the role of the *UPS* gene family in potato, we analyzed the transcriptome data and generated a heatmap employing TBtools (Figure 7). The results demonstrated that the majority of the *StUPS* genes were highly expressed in leaves and shoots relative to the low expression in roots. In contrast, the *StUPS7* gene was highly expressed in potato roots but less so in potato leaves. In addition, the expression of most of the *StUPS* genes was significantly up-regulated under abiotic stress, especially under drought (mannitol-mimicking drought stress) and salt stress. Among these genes, *StUPS7* expression did not change significantly under drought and IAA stress. High-temperature and BAP treatments had a slight effect on StUPS expression.

### 3.8. Expression Verification of Potato UPS Genes

**Figure 8:**
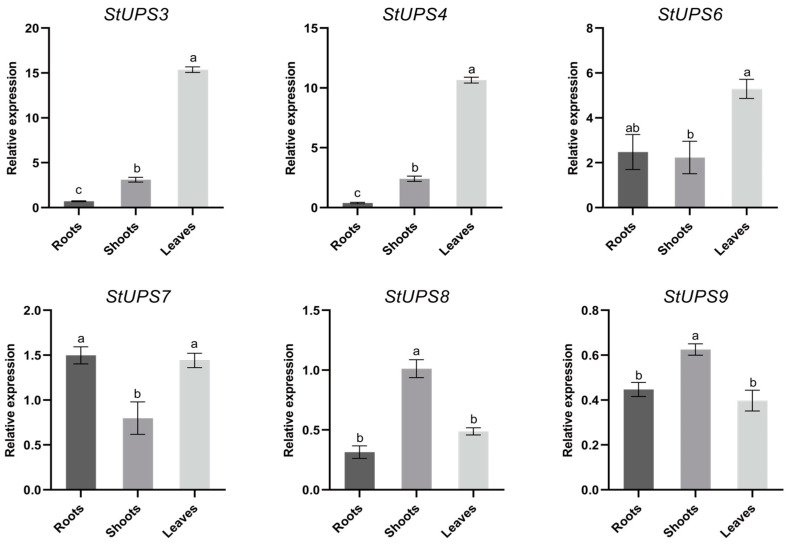
Expression analysis of six *StUPS* genes selected in roots, shoots, and leaves. Analyzing three measurements to obtain error bars. Different letters above bars indicate significant differences between tissues (*p* < 0.05).

**Figure 9:**
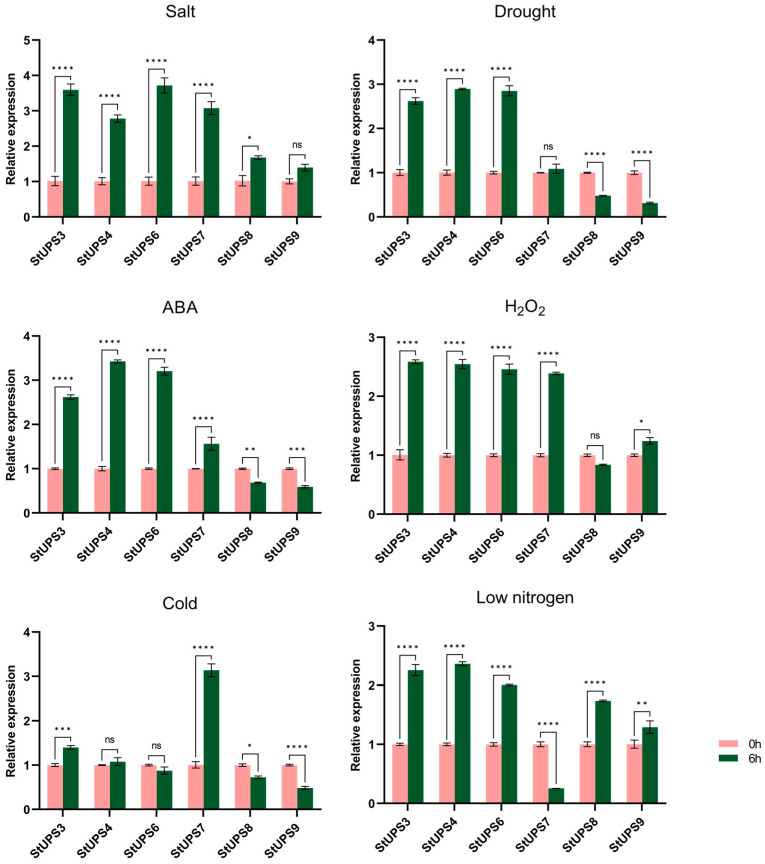
Expression analysis of six *StUPS* genes under abiotic stress. Analyzing three measurements to obtain error bars. Asterisks above the bars indicate significant difference between treatments, while ns indicates no significant difference (*p* < 0.05).

To further validate the function of the UPS family, six genes (*StUPS3*, *StUPS4*, *StUPS6*, *StUPS7*, *StUPS8*, and *StUPS9*) were screened for strong responses to abiotic stress based on the clustered heatmap. One control group and six stress-treated groups were established to investigate the expression patterns in various tissues and under abiotic stresses, and the results are displayed in Figure 8 and Figure 9. Three genes, *StUPS3*, *StUPS4,* and *StUPS6*, were strongly expressed in roots, whereas *StUPS7* was highly expressed in roots and leaves but was expressed at lower levels in shoots. Two genes, *StUPS8* and *StUPS9*, were most highly expressed in shoots, and there was no significant difference in expression in either roots or leaves (Figure 8).

In addition, we analyzed qPCR data to investigate the function of *StUPS* genes in response to abiotic stress (Figure 9). Under salt treatment, all six *StUPS* genes were up-regulated at 6 h compared to 0 h, and with the exception of *StUPS9*, the change was statistically significant. Under drought treatment, *StUPS3*, *StUPS4*, and *StUPS6* were significantly up-regulated at 6 h; *StUPS7* expression levels were elevated, but the difference was not significant. In addition, *StUPS8* and *StUPS9* were significantly down-regulated at 6 h. At 6 h after ABA treatment, *StUPS3*, *StUPS4*, *StUPS6*, and *StUPS7* were significantly up-regulated, but StUPS8 and StUPS9 were significantly down-regulated. Under H_2_O_2_ treatment, all *StUPS* genes were significantly up-regulated except for *StUPS8*, whose expression decreased at 6 h, but the difference was not significant. Under cold stress conditions, the expression of StUPS3, *StUPS4*, and *StUPS7* was significantly up-regulated at 6 h of treatment, whereas the expression of *StUPS6*, *StUPS8*, and *StUPS9* was significantly down-regulated at 6 h compared with 0 h. Under low-nitrogen treatment, all *StUPS* genes were significantly up-regulated at 6 h, except *StUPS7*, which was significantly down-regulated at 6 h of treatment.

## 4. Discussion

Due to their relatively high nitrogen-to-carbon ratio, ureides are an efficient form of nitrogen storage and translocation [14]. UPS proteins have been extensively studied in legumes and *Arabidopsis* [3,4,6,7,18]. In both nitrogen-fixing and non-nitrogen-fixing legumes, the manipulation of UPS expression results in impaired allantoin and allantoate translocation from roots to source leaves [17,35]. For example, *OsUPS1* overexpression results in enhanced accumulation of allantoin and total free amino acids [36]. However, studies on the UPS family in potato, an important food crop, have not been reported. Therefore, we identified 10 *StUPS* genes through our research and analyzed the physicochemical properties and subcellular localization of their proteins. StUPS proteins are all hydrophobic and stabilizing proteins except for StUPS1. Assessing the subcellular localization of transporters is crucial to understand their molecular function. Previous studies have demonstrated that UPS is predominantly localized in the plasma membrane [7,35,36]. Some studies have also demonstrated that UPS localization is more complex and that UPS also localizes in the endoplasmic reticulum, Golgi apparatus, and other sites [18]. The projected subcellular localization results in this study revealed that StUPS4, StUPS9, and StUPS10 were localized in the plasma membrane; StUPS3, StUPS5, and StUPS6 were localized in the vacuole; StUPS1 and StUPS8 were localized extracellularly; and StUPS2 and StUPS7 were localized at the endoplasmic reticulum and chloroplasts, respectively. In *Arabidopsis*, AtUPS5L and AtUPS5S are localized in the endoplasmic reticulum membrane, through which allantoin can enter the intracellular membrane system [18]. In addition, unique genes for allantoin degradation (allantoinase or *AtALN*) are localized in the endoplasmic reticulum lumen [37]. Under non-stress conditions, AtUPS5L and AtUPS5S may be involved in the allantoin degradation nutrient cycle. Under stress, *AtUPS5L* and *AtUPS5S* may be essential genes for the outward secretion of allantoin through vesicles, allowing for the transport of allantoin from roots to shoots [18].

Genes replicate to generate two or more copies [38], which encode similar protein products and have comparable structures and functions. As a result, similar gene family members can be discovered in numerous species, and recent studies have shown that there are 13 *UPS* genes in wheat [39] and 3 in rice [15]. During gene evolution, the duplication or loss of genes might result in an unequal number of gene family members [40,41]. According to the phylogenetic tree (Figure 2), the majority of the UPS proteins exhibited great similarity across two distinct species, and the 10 StUPS proteins were categorized into four groups. It is hypothesized that UPSs are highly conserved across species and existed before the isolation of monocotyledons. Next, we acquired the conserved motifs of the 10 StUPSs and discovered that the distribution of these motifs varied between groups but was comparable within them. As an example, StUPS4, StUPS6, StUPS9, and StUPS10 contain motif 6 and motif 9, and the four StUPS proteins belong to Group C. Common motifs in genes typically result in functional redundancy, whereas particular motifs can lead to functional divergence [42]. In addition, gene organization patterns and exon structures play a significant role in understanding the evolutionary mechanisms of a gene family [37,38]. Figure 3 shows that all *StUPS* genes possess untranslated regions, and the number of exons spans from 1 to 7. There is an association between gene structures and motifs, which supports the categorization of StUPSs.

In Figure 4, the 10 *StUPS* genes are distributed on three chromosomes. In the evolution of plants, gene duplication is a crucial process that produces new genes with unique roles [43]. The three primary modes of plant evolution are tandem duplication, translocation events, and fragment duplication; gene family expansion typically proceeds via the first two modes [44,45]. The colinear relationship between the *StUPS* gene pairs is depicted in Figure 5, and although no tandem duplication was discovered, one pair of segmental duplications was reported. Our results indicate that the aforementioned gene pairs were produced by genome-wide duplication. We detected six pairs of collinearity between potato and tomato, and four pairs of collinearity between potato and *Arabidopsis*, which did not differ significantly from each other (Figure 6). This finding is consistent with species affinities and indicates that the *UPS* gene families in potato and *Arabidopsis* are highly related during the evolutionary process. As a consequence, we hypothesize that they may form similar functions.

The expression patterns of *UPS* genes in various tissues have been reported in several species [15,18,36]. A heatmap (Figure 7) revealed that the remaining nine *StUPS* genes were strongly expressed in leaves, except for *StUPS7*, which was substantially expressed in roots. To investigate the potential role of *StUPSs* in potato development, we next performed RT–qPCR experiments to examine their expression levels in different tissues. According to the results presented in Figure 8, *StUPS3*, *StUPS4*, and *StUPS6* were strongly expressed in roots; *StUPS7* was substantially expressed in roots and leaves but was expressed at lower levels in shoots. Two genes, *StUPS8* and *StUPS9*, exhibited the highest expression in shoots, and there was no significant difference in expression in either roots or leaves. The expression of the *StUPS* genes in tissues detected through RT–qPCR, as described above, was generally consistent with the transcriptome data. The expression of various StUPS genes in potato tissues differed, indicating that *StUPS* genes perform distinct functions in potato growth and development.

Abiotic stresses are the primary cause of yield losses globally [46]. Breeding resilient crops may significantly improve crop quality and production. Ureides, including allantoin and allantoate, play a vital role in nitrogen metabolism and stress responses in nitrogen-fixing and non-nitrogen-fixing plants. Ureides are generally synthesized in roots, and UPS is involved in the transport of ureides from roots to aboveground organs, which has been investigated in *Arabidopsis* and legumes [3,5,7,10]. *PvUPS1* overexpression in soybean increases allantoin and allantoate output from rhizomes, which improves the nitrogen supply to shoots [12]. In rice, allantoin in *OsUPS1*-overexpressing lines accumulates significantly in root, stem, and leaf tissues. In contrast, *OsUPS1^RNAi^*-silencing lines exhibit lower allantoin levels in identical tissues, with the exception of roots, where allantoin is observed to accumulate. Therefore, researchers have concluded that *OsUPS1* is the main gene responsible for allantoin distribution in rice. It influences plant growth and development by driving the accumulation of allantoin in reservoir tissues, which can then be used to overcome adversity during low-nitrogen stress [36]. In recent years, allantoin has garnered interest from the scientific community, as various metabolomic studies indicated that this compound accumulates in a broad spectrum of plant species under drought [47,48,49,50,51,52], high-salt [53,54,55], cold [56], and sulfate starvation [57] conditions. Studies on *Arabidopsis* and rice have shown that UPS affects plant stress tolerance by mediating the accumulation of allantoin [21,36]. High levels of allantoin prevent damage induced by salt stress [21,58,59]. Investigations on wheat have shown that the accumulation of allantoin under drought conditions relieves the strain in the GS-GOGAT cycle, thus preventing the accumulation of toxic levels of NH_4_^+^ and possible N losses due to volatilization [14].

In our study, the expression of most *StUPS* genes was considerably up-regulated under abiotic stress, which is generally consistent with results in *Arabidopsis* [18]. Under drought, ABA, and cold stress, the expression of both *StUPS8* and *StUPS9* genes was significantly down-regulated at 6 h of treatment compared to 0 h. It is anticipated that the accumulation of ureides may cause feedback regulation of the two genes, resulting in their expression being reduced. Under salt, drought, ABA, H_2_O_2,_ and low-nitrogen treatments, the trends of *StUPS3*, *StUPS4*, and *StUPS6* expression were similar, and it is hypothesized that they exhibit the same function in response to stress. In addition, the trends of *StUPS8* and *StUPS9* were similar under salt, drought, ABA, cold, and low-nitrogen stresses. Under cold stress, *StUPS7* showed the most pronounced and significant change compared with the other *StUPS* genes, and was hypothesized to play a dominant role under cold stress. However, under low-nitrogen stress treatment, *StUPS7* showed the opposite trend. The findings presented above reveal that *StUPS* family genes are critical for potato growth and development, as well as for sensitivity to abiotic stressors.

## 5. Conclusions

In summary, we identified 10 UPS family members at the potato genome-wide level and categorized them into four groups. In addition, our results indicate high homology between *Arabidopsis* and potato. Moreover, the results of gene localization on chromosomes demonstrate that *StUPSs* are located on three chromosomes, and one pair of repetitive fragments is identified by covariance analysis. Subsequent RT–qPCR experiments verified that the six chosen *StUPS* genes exhibit distinct expression patterns, suggesting that they participate in the regulation of salt, drought, ABA, H_2_O_2_, cold, and low-nitrogen stress responses. During the evolutionary process, *StUPS* genes are highly conserved. These genes are engaged in the regulation of potato growth and development, and they also play a significant role in the response to adversity in potato plants. We have a preliminary understanding of the functions of the *StUPS* gene family members under abiotic stresses that provides a solid foundation for enhancing the genetic breeding of potato plants via genetic engineering.

## Figures and Tables

**Table 1:** Location and physicochemical properties of potato UPS family.

ID	Sequence ID	Number of Amino Acid	Molecular Weight	pI	Instability Index	Aliphatic Index	Grand Average of Hydropathicity	Localization Prediction
PGSC0003DMT400025459	StUPS1	59	6671.11	10.55	40.63	108.98	0.731	Extracellular
PGSC0003DMT400025460	StUPS2	65	6942.19	10.16	39.34	96.15	0.598	Endoplasmic reticulum
PGSC0003DMT400025462	StUPS3	319	34,322.81	9.09	33.03	103.1	0.443	Vacuole
PGSC0003DMT400025463	StUPS4	407	44,147.34	8.87	32.33	105.5	0.465	Plasma membrane
PGSC0003DMT400025464	StUPS5	319	34,322.81	9.09	33.03	103.1	0.443	Vacuole
PGSC0003DMT400025465	StUPS6	233	25,250.13	6.13	32.77	106.78	0.302	Vacuole
PGSC0003DMT400045883	StUPS7	95	10,056.74	9.43	30.23	96.74	0.537	Chloroplast
PGSC0003DMT400072929	StUPS8	113	12,247.4	9.84	35.1	99.47	0.477	Extracellular
PGSC0003DMT400072930	StUPS9	406	44,175.16	8.04	31.08	95.44	0.385	Plasma membrane
PGSC0003DMT400072931	StUPS10	406	44,175.16	8.04	31.08	95.44	0.385	Plasma membrane

## Data Availability

The data presented in this study are available within the article.

## Supplementary Materials

The following supporting information can be downloaded at: https://www.mdpi.com/article/10.3390/genes15070870/s1, Table S1: Different stress treatments, Table S2: StUPS gene primer selection.
